# Breast Cancer Patients’ Experiences with Online Group-Based Physical Exercise in a COVID-19 Context: A Focus Group Study

**DOI:** 10.3390/jpm12030356

**Published:** 2022-02-26

**Authors:** Maria Elena Garcia-Roca, Miguel Rodriguez-Arrastia, Carmen Ropero-Padilla, Carlos Hernando Domingo, Ana Folch-Ayora, Maria Dolores Temprado-Albalat, Ana Boldo-Roda, Eladio Collado-Boira

**Affiliations:** 1 Faculty of Health Sciences, Pre-Department of Nursing, Jaume I University, 12071 Castello de la Plana, Spain; garciroc@uji.es (M.E.G.-R.); arrastia@uji.es (M.R.-A.); afolch@uji.es (A.F.-A.); colladoe@uji.es (E.C.-B.); 2 Research Group CYS, Faculty of Health Sciences, Jaume I University, 12071 Castello de la Plana, Spain; 3 Sport Service, Jaume I University, 12071 Castello de la Plana, Spain; hernando@uji.es; 4 Department of Education and Specifics Didactics, Jaume I University, 12071 Castello de la Plana, Spain; 5 Department of Medicine, Cardenal Herrera-CEU, CEU Universities, 46115 Valencia, Spain; maria.temprado@uch.ceu.es; 6 Department of Gynaecology and Obstetrics, La Plana University Hospital, 12540 Vila-Real, Spain; boldo_ana@gva.es; 7 Fisabio Foundation, 46020 Valencia, Spain

**Keywords:** adverse effects, breast cancer, COVID-19, exercise, focus groups, women

## Abstract

In patients with breast cancer, physical exercise reduces the toxicity of treatment; however, this physical exercise must meet a set of criteria, such as being guided by knowledgeable instructors. Thus, the aim of this study was to explore the perceptions of female breast cancer patients regarding the impact of an online physical exercise programme in the context of the COVID-19 pandemic. Nineteen female breast cancer patients participated in four focus group interviews as part of a qualitative study using a thematic analysis between December 2020 and May 2021. Three major themes emerged: “Experiences and perceptions of online physical exercise with breast cancer”; “Incorporating exercise-based activity for cancer-related side effects”; and “Increasing self-esteem and empowerment”. Online, live-streamed, and supervised group activities help breast cancer patients engage and prevent the recurrence of cancer-related side effects, as well as to control COVID-19-related fear and provide an alternative to promote mental health-related quality of life.

## 1. Introduction

Female breast cancer is one of the most prevalent malignancies worldwide, accounting for 25% of all diagnosed cancers [1]. Currently, the most effective form of treatment is surgery combined with complementary local therapies, such as radiotherapy, or systemic treatments, such as chemotherapy, hormonal therapy, and targeted therapies [2]. Although the survival rates in women diagnosed with cancer continue to increase, the treatments that are administered to curb disease progression carry side effects that affect quality of life [3]. Surgery can trigger discomfort at the surgical site, the loss of muscle mass due to immobility, and lymphedema secondary to lymph node resection [4], while systemic treatments can produce long-term side effects, such as cardiac toxicity, osteoporosis, and joint pain, among others [5].

One strategy to control the side effects of these treatments is the prescription of physical exercise [6]. Several studies have shown how physical exercise reduces the toxicity of treatment in patients with breast cancer, improving physiological and functional parameters as well as quality of life [7,8,9]. As supported by the International Agency for Research on Cancer (IARC), a physical exercise regimen can be routinely prescribed to women with breast cancer both during treatment and afterwards, although they must meet a series of characteristics [8,10]. The sessions must be guided by knowledgeable instructors who are capable of offering personalised exercise programmes with respect to the type of tumour, the treatment being administered, and the individual characteristics of each patient [11].

Strict isolation and social distancing measures were implemented in response to the COVID-19 pandemic [12,13,14], particularly affecting vulnerable populations. In the case of cancer patients, cutbacks were made to the prescription of guided and supervised physical exercise, and certain clinical procedures were suspended or delayed [15,16]. Some studies have shown the importance of designing group-based physical activity programmes to promote mental health and resilience in cancer survivors [17,18]. However, studies on breast cancer patients undergoing active treatment have focused on the prescription of physical exercise in person both individually and in small groups [19,20]. Despite the demonstrated effects of physical exercise, to our knowledge no study has qualitatively researched the use of an online group exercise programme with adapted and individualised follow-up in breast cancer patients undergoing active treatment during the pandemic.

## 2. Materials and Methods

### 2.1. Aim

The objective of this study was to explore the perceptions of female breast cancer patients undergoing treatment regarding the impact of an online physical exercise programme on quality of life in the complex context of the COVID-19 pandemic.

### 2.2. Design

To gain an in-depth understanding of breast cancer patients’ experience towards online group-based physical exercise during the COVID-19 pandemic, the study adopted a qualitative approach using focus groups interviews [21]. This study was performed at Jaume I University with female breast cancer patients undergoing cancer treatment between December 2020 and May 2021.

### 2.3. Participants

A purposive sampling approach was used at Jaume I University. The selection criteria included female patients who: (i) were 18 or older; (ii) were diagnosed with breast cancer (ICD-10: C50 or ICD-9: 174, 175, and V10.3) undergoing cancer treatment (chemotherapy, hormone therapy, or immunotherapy); (iii) participated in bi-weekly online physical training in streaming offered by M.E.G.-R. and supervised by their oncology team for at least 6 months; and (iv) prior to taking part in this research agreed to provide written informed consent.

### 2.4. Procedure

An online physical exercise program was designed to increase strength, joint mobility, and cardiovascular capacity, with the purpose of improving quality of life by increasing patients’ tolerance to cancer treatments. This programme, delivered via streaming as a result of the pandemic, was designed by a multidisciplinary team (two graduates in physical activity and sport with a specialisation on oncology, two medical oncologists, two specialists in gynaecological and breast surgery, two doctors in health sciences and one psycho-oncologist) to allow participants to benefit from physical exercise supervised and directed by a specialised professional.

A total of 25 women participated in the physical exercise program and were divided into two homogeneous groups based on age and physical fitness variables, the values of which were obtained from baseline measurements. Aerobic capacity (walking test), strength (stand chair, hand grip, and squat jump test), flexibility (sit and reach test), and daily physical exercise were the variables studied (continuous monitoring with accelerometery for 7 days). Each of these groups was further divided into two groups per schedule, yielding two groups of eight patients, one group of six patients, and one group of five patients. The frequency of the programme was two 60 min sessions per week. Seventy-six percent of the participants (*n* = 19) completed the entire program, while 24% (*n* = 6) dropped out; eight percent of the participants (*n* = 2) dropped out due to disease progression, another 8% (*n* = 2) due to scheduled surgery, and the remaining participants (*n* = 2, 8%) gave no reason. The women who completed the program (*n* = 19) attended 95% of the scheduled sessions.

The physical exercise program consisted of 48 sessions spread out over six months. The sessions were split into three sections. The first ten minutes of the warm-up were spent doing joint mobility exercises. The main part was 40 min of strength-resistance work (e.g., squats, front and side lunges, sit-ups, biceps, triceps, etc.). The final 10 min segment included flexibility exercises for the large muscle groups. At the end of the session, a fatigue scale (Borg scale) was applied, and the intensity of the subsequent sessions was programmed based on the percentage of fatigue reached. The intensity was adapted to reach fatigue values of 60 to 75% in the two subgroups that obtained better values of the physical variables in the baseline measurement, while the two subgroups with lower values worked at intensities that resulted in fatigue values of 50 to 70%.

### 2.5. Data Collection

Four focus group interviews (FGs), with 4 to 5 female patients participating in each interview, were carried out. Researchers developed and agreed on an interview protocol designed to encourage participants to give detailed responses (Table S1). All the interviews were conducted at Jaume I University by two researchers experienced in conducting qualitative interviews with oncology patients, and in accordance with COVID-19 capacity and safety protocols. Each interview lasted 40 to 60 min and the audio was digitally recorded. Data collection was continuously analysed until data saturation was reached. To ensure the anonymity of the participants in the transcription of the interviews, the letters “G” (group) and “P” (participants) were employed, along with the participant number. Prior to the analysis, the participants were given the option of reviewing the transcripts.

### 2.6. Data Analysis

**Figure 1:**
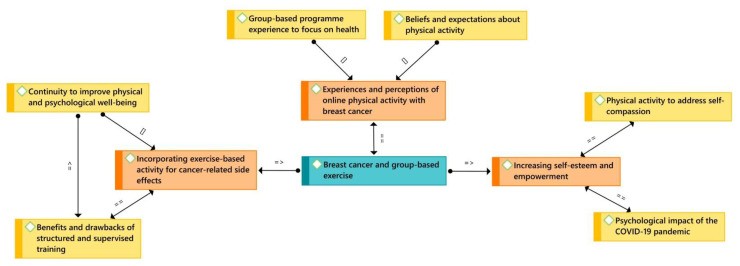
Conceptual map based on female breast cancer patients’ experiences with physical exercise during the COVID-19 pandemic ([]—is part of; = =—is associated with; = >—is a cause of).

All interviews were transcribed and anonymised prior to their analysis with the ATLAS.ti 9 software. In this regard, a thematic analysis elaborated by Braun and Clarke [22] was used, which contains the following steps: (i) data familiarisation by reading all transcripts repeatedly, (ii) arranging pertinent data into significant codes, and (iii) grouping the codes into possible themes. Later, (iv) thematic validity is confirmed by reading all codes and the entire data set, (v) before defining and naming them, and (vi) elaborating a final report (Figure 1).

### 2.7. Ethical Considerations

The Ethics Committee of Jaume I University approved the study, which adhered to all of the principles of the Helsinki Declaration and its subsequent revisions. Before conducting the study, participants provided informed consent and the data collection design ensured confidentiality and anonymity. Participants were also notified that their experiences, opinions, and perspectives would not affect their academic grades.

### 2.8. Rigour

This study was developed using the recommendations of the consolidated criteria for reporting qualitative research (COREQ) [23]. Two authors (M.E.G.-R. and M.R.-A.) individually analysed written descriptions before meeting together to contrast, associate, and debate the emerging themes in order to attain conformability. In the event of a discrepancy, a third researcher (C.R.-P.) was consulted to ensure that the collected data were reliable and consistent.

## 3. Results

### 3.1. Participant Characteristics

A total of 19 female breast cancer patients participated in four FGs conducted in May 2021. The participants’ average age was 49.2 years old (SD = 9.2), with a range of 28 to 66. The most frequent tumour stage was stage II with 63.1% (*n* = 12) of the participants. A total of 84.2% of the participants (*n* = 16) received surgery and 42.1% (*n* = 8) received chemotherapy (doxorubicin and cyclophosphamide) in addition to radiotherapy. The qualitative analysis disclosed three major themes, which are presented in Table 1.

### 3.2. Theme 1: Experiences and Perceptions of Online Physical Exercise with Breast Cancer

This first theme addresses two sub-themes and depicts the participants’ expectations and experiences with an online group-based physical exercise programme while undergoing cancer treatment during the COVID-19 pandemic. In particular, our data revealed the potential effects of this group-based physical exercise programme on patients’ well-being during cancer treatment.

#### 3.2.1. Sub-Theme 1.1: Beliefs and Expectations about Physical Exercise

Physical exercise during cancer treatment was something that some participants had not previously considered to be beneficial to the cancer disease process. In fact, the participants emphasised the significance of the breast oncology team’s recommendation of physical exercise while undergoing cancer treatment in order to feel it was a part of the treatment. On the other hand, others were convinced that sports were beneficial for them, but government restrictions imposed as a result of the COVID-19 pandemic forced them to forego some of their sports activities:

“*I wouldn’t have considered sports to be a core element of my treatment, but I believe it also has an impact on who makes the recommendation. You sort of say, “hey, this looks like a treatment”. True, it is a different kind of treatment, but when a health professional says so, it is because it is going to help me. Being honest, I take it into account more than if someone else who isn’t a healthcare professional says it*”

G4-P4

“*I have always been active, but since being diagnosed with breast cancer, I have become more cautious in all aspects of my life, especially with the COVID-19... I couldn’t risk going to the gym, so when I heard about the online group, I thought it was perfect because it was about much more than just doing sport by my own; it was about being able to speak with other people who are dealing with the same thing as you, a two-for-one, sport and group therapy with people who have your same issues*”

G2-P3

#### 3.2.2. Sub-Theme 1.2: A Group-Based Exercise Programme Experience to Focus on Health Not Illness

Likewise, the majority of the participants stated that taking part in a well-planned and organised online group-based physical exercise programme, as well as having a knowledgeable instructor and being supervised, was an important factor in feeling motivated, being person-centred cared, and being able to socialise and empathise with the other women:

“*It is well worth it. I genuinely think that sport is very essential, but I also believe that the instructor’s work is important, when she said to us, “come on, you are doing very well”; aside from the exercise, it is the motivation that you had during that time, it was a therapy, it was supervised, and there was feedback of what we were doing. There is a person who is following you, who is following you up*”

G1-P1

“*This exercise programme has been a huge lifesaving boat for me that allowed me to interact with others who gave me a lot and helped me not to make the day-to-day life of being alone so challenging. I had my time of sociability, my time to talk with the instructor, mitigating all of the negative aspects of the pandemic*”

G3-P2

### 3.3. Theme 2: Incorporating Exercise-Based Activity for Cancer-Related Side Effects

This second theme identifies the importance of participating in a group-based exercise programme during cancer treatment for patients’ biopsychosocial well-being, as well as how this experience helps them promote and engage in physical exercise in their daily lives.

#### 3.3.1. Sub-Theme 2.1: Benefits and Drawbacks of Structured and Supervised Training

Many of the participants emphasised that this group-based physical exercise experience had an impact on every aspect of their lives, but most notably on the relief of cancer treatment side effects, such as pain, tiredness, motivation, and attitude and behaviour problems, among others:

“*I used to have to go to bed because I was in so much agony. “I’m still and I’m exhausted!” I cried. The next day, I was unable to do anything. However, you get up, warm up, and exercise at the start of the supervised exercise programme... Of course, you get tired, but you rest a little, and the next day, you feel great. I’ve been performing the exercise training for months, and I’ve found that the pain returns after 2–3 days of not doing it, so I have to keep doing it!*”

G2-P1

“*My joints hurt when I first started the training programme, I had more hot flashes, and I could hardly sleep. Yet, I have almost no hot flashes, no joint pain, and I sleep a lot better with this treatment and exercise routine. My quality of life has improved significantly, so I intend to continue the supervised programme*”

G1-P5

Despite the overall benefits of online group-based exercise training, the lack of physical interaction and face-to-face activities was the most significant disadvantage for participants:

“*I have missed being able to get together and spend some time conversing with others after the training class. The COVID-19 pandemic made me realise how much I need physical contact; I enjoy interacting with others and having a chit-chat every now and then*”

G3-P4

“*Not seeing each other, not laughing when we are knackered and soaked in sweat... Definitely, the main drawback for me was that there is no physical contact*”

G4-P2

#### 3.3.2. Sub-Theme 2.2: Continuity to Improve Physical and Psychological Well-Being

One of the aspects most frequently mentioned by participants was how their physical and psychological well-being improved during this supervised group-based physical exercise experience, prompting them to introduce exercise into their daily routine as any other important aspect of life and ensure adherence to physical exercise:

“*I said at the beginning “What a hassle, but well… Let’s see if I can make it through the next six months”. Now, when we are not with the instructor, I am working hard as well. I will never stop because the difference between how I used to be and how I am now is breathtaking. I feel strong and, most importantly, confident since my biggest anxiety was with my bones, and now that I have the muscles to support everything, it offers me a lot of peace of mind*”

G3-P1

“*Although there are days when I am exhausted because my medication or have things to do, it is true that I have made physical exercise a part of my daily routine. My quality of life has drastically improved, it is like my treatment now... And it has influenced others to support me. We are moving like chess pieces so this, which is a priority for me, can keep going*”

G1-P2

### 3.4. Theme 3: Increasing Self-Esteem and Empowerment

The last theme offers some insight into the importance of group-based physical exercise in which all the participants share similar characteristics, allowing them to relate their experiences with the cancer disease and treatment process and support each other. Interestingly, the participants also identified other perks, such as how participating in an online physical exercise training environment made them feel safer from infection during the COVID-19 pandemic.

#### 3.4.1. Sub-Theme 3.1: Physical Exercise to Address Self-Compassion

A large number of participants described how sharing this exercise programme with other women experiencing similar disease symptoms made them feel accompanied, understood, and stronger in their fight against cancer:

“*I believe that in this disease, the support between each other is critical. I believe that when we are all in the same situation, the amount of empathy that can be awakened among us increases enormously. I don’t feel your friends or family truly comprehend what is happening to you. When we talk, you are never delighted that the other person is also hurting, but you are soothed by the notion that your bones hurt because we all do*”

G4-P5

“*Doing the exercise programme and deciding to form a group in which we could express our feelings, what was going on in our lives, and even laughed at ourselves. That was hugely helpful for my self-esteem since we could normalise the situation. We chatted about what was going on with us, our fears, and our rejection of our own image… We encouraged one another, we supported each other. Joining the group has been a liberating experience for me*”

G2-P4

#### 3.4.2. Sub-Theme 3.2: Psychological Impact of the COVID-19 Pandemic

One of the key topics was the online approach for the group-based physical exercise programme in which the women participated that allowed them to feel fearless and safe from infection, despite the immunosuppression induced by cancer treatment. Although they were particularly concerned about COVID-19 infection since it would halt their cancer treatment, the online fitness programme appeared to be a useful alternative for balancing physical exercise with family, job, and other responsibilities:

“*The COVID-19 horrified me; if you got it, your treatment would be delayed, which was unthinkable to me. To be honest, I felt really alone, but I started with this online exercise training here and gradually improved. You can see that there are more people like you; thus, you must participate in activities and keep moving forward. If it had been in person, I would not have gone out of fear, but rather out of caution*”

G1-P4

“*I find the fact that it was done in streaming was extremely positive because I feel that all of us were worried of the COVID-19 during our chemotherapy or radiotherapy process. Between that fear and personal circumstances such as kids, jobs, travel and time constraints, it was a positive change to do it in streaming*”

G2-P2

## 4. Discussion

This study aimed to explore breast cancer patients’ perceptions towards the impact of online physical exercise in their quality of life whilst receiving cancer treatment through the understanding of their experiences in a complex scenario as a consequence of the COVID-19 pandemic. Following the analysis of our findings, it was found that almost all participants reported a revamped engagement, motivation, and self-esteem with the use of the online supervised group-based exercise training. Although the importance of exercise for women during breast cancer survivorship has been discussed [24,25,26], this study yielded some interesting findings regarding the impact of an online group-based exercise programme to optimise adherence. To the best of our knowledge, this is the first study to explore female breast cancer patients’ experiences regarding barriers and motivators to physical exercise during the COVID-19 pandemic whilst receiving treatment for breast cancer from a qualitative perspective.

Our findings, similar to those of face-to-face training programmes, showed that structured and supervised online exercise training is a safe strategy for breast cancer patients, offering improvements in fitness, physiological and psychological functions, and cancer-related side effects [25,27]. Although female breast cancer patients are generally motivated to stay healthy, inaccurate beliefs and misperceptions about physical exercise appear to be a barrier to participating in exercise [28]. Indeed, breast cancer patients’ pre-existing beliefs, values and attitudes towards physical exercise tend to be similar to those affecting the general population, and a lack of accurate information about safe exercise influences their decisions regarding exercise, despite its well-documented benefits [29]. One possible explanation for this could be the manner in which physical exercise and treatment regimens are offered to women and relatives, which is sometimes associated with the non-essential aspects of treatments [30,31].

In contrast to recent studies [32,33], most participants believed that the supervised group-based physical training programme improved their energy levels and well-being. For our participants, group dynamics could be as effective as an individual exercise in improving their quality of life, as they are able to engage socially whilst training. While the level of physical exercise experience varied among our participants, having a knowledgeable instructor along with their breast oncology team helped in tailoring appropriate physical group-based activities [6]. Certainly, the importance of group-based exercise in encouraging continuity and promoting peer social support with others was the most positive aspect highlighted in our findings [26,34,35]. As our participants reported, these online and supervised group-based physical activities provided a safe and caring environment during a time of mobility restrictions due to the COVID-19 pandemic, and assisted in overcoming previously reported hurdles, such as bad weather, costs, or time constraints [36]. These findings further support to the ideas of self-determination theory and achievement goal theory [37,38], which advocate for the use of group-motivated cognitive-behavioural interventions, such as self-monitoring, goal setting, support, feedback, and relapse intervention, and suggest that a task-oriented environment can improve motivation and adherence [39]. Furthermore, recent research indicates that interventions which foster a positive, task-oriented, and caring environment during breast cancer treatment result in increased motivation, positive attitudes towards exercise, and optimism among patients [40]. An implication of this emotional positive experience is the possibility that a caring and supervised training exercise may help breast cancer patients improve their psychological adjustment via self-compassion [41]. Short-term and long-term group-based physical exercise programmes have been shown to reduce anxiety, depression, or body image disturbance, and may therefore constitute a good opportunity to foster self-compassion cultivation in patients undergoing treatment for breast cancer [25,42].

That being said, our participants highlighted novel and specific psychological implications of the COVID-19 pandemic on their quality of life. Aside from previously documented cancer-related side effects, such as upper extremity issues, fatigue, pain, depression, or body image disturbance [24,43], fear was the most common concern among participants. This fear was clearly driven by the possibility of delays or even disruptions in cancer care as a result of the relative risks of COVID-19 exposure [44]. In this sense, not only did online and supervised group-based activities assist patients in engaging and preventing the recurrence of cancer-related side effects, but they also controlled COVID-19-related fear and provided an alternative to promote mental health-related quality of life. This is in accordance with earlier observations, which showed that online, web-based, and digital interventions have potential for physical exercise promotion among cancer patients [45,46], though a more sustained and direct instructor-to-group approach, as suggested by our participants, would be required.

Nevertheless, there are certain limitations that should be considered. To date, there is limited research investigating the impact of physical exercise on female breast cancer patients receiving cancer treatment, which has limited the discussion of our findings. Future research should explore the experiences and perceptions of instructors in order to gain a deeper understanding of the use of group-based physical exercise programmes, as well as possible alternatives to the identified drawbacks. Moreover, these findings warrant further discussion, for example, surrounding the inclusion of prescribed exercise programmes into cancer care treatments or the use of group-based programmes with other types of cancers.

## 5. Conclusions

Our findings indicate that group-based physical exercise has the potential not only to instil self-esteem and address self-compassion, but also empower women to be confident during their treatment and prevent cancer-related side effects. Given the physical and psychological health benefits of regular physical exercise for this population, promoting physical activity in women diagnosed with and being treated for breast cancer must be an essential public health priority. Cancer care teams and experienced instructors may facilitate person- and cancer-centred care in a timely, accurate, and tailored manner, whereby providing a caring and group-based supportive climate may offer a safe environment in which breast cancer patients can feel comfortable, engage socially, and acquire exercise adherence. Thus, online and live-streamed physical exercise programmes are a viable approach for promoting physical and mental well-being among female breast cancer patients while they are undergoing treatment.

## Figures and Tables

**Table 1:** Themes, sub-themes, and representative quotes.

Main Themes	Sub-Themes	Representative Quotes
Experiences and perceptions of online physical exercise with breast cancer	Beliefs and expectations about physical exercise	*“Sport has provided me with a level of support that I could never have imagined. When they asked me, “do you want to be a part of this exercise group?”, I did not hesitate. I needed it emotionally and physically. I felt somehow saved, and when I started, it was fantastic”* G1-P3
		*“I knew I wanted to keep doing sports because I know it gives me both emotional and physical stability. Having to leave my paddle team in order to lock myself away at home and do nothing that motivated me has made joining this group a life-changing experience”* G4-P1
	A group-based exercise programme to focus on health not illness	*“I would not have done it on my own if I hadn’t been in a group. This has been highly helpful to me; without it, I believe I would have collapsed somehow”* G3-P5
		*“Today, the best way for me is to do it as a group. Because looking at the one who does better stimulates me and helps me strive to achieve higher goals, because you feel accompanied and enriched by the others”* G2-P3
Incorporating exercise-based activity for cancer-related side effects	Benefits and drawbacks of structured and supervised training	*“With the pain and fatigue, you think you can’t do it because you are too weak. However, you make the extra effort with this training and it gives you the energy to keep going and not lie in bed all day”* G3-P4
		*“There has been a complete before and after. I believe this training has helped me in avoiding the “I can’t” circle. It has given me the confidence to say, “yes, I can, I can do this, and I will get it back”* G4-P2
	Continuity to improve physical and psychological well-being	*“I now do exercise every day, and the days I don’t, I miss it. I didn’t go out much before, and I still don’t go out much now, but I feel a lot better. I don’t think about anything negative, and I rarely consider cancer. I am thrilled and grateful”* G1-P5
		*“When I prepare my weekly schedule, I include things like “this time to walk to the mountain, this time for the exercises...”I now include it in my plans, which I never used to do before”* G3-P3
Increasing self-esteem and empowerment	Physical exercise to address self-compassion	*“Being able to virtually interact with a group with whom you can share experiences, the impact of seeing yourself hairless... I believe that seeing that we are all going through this together, exercising together, always helps psychologically. It supports you in assimilating of the entire situation you are experiencing”* G2-P4
		*“I felt identified, as though I wasn’t the only one who felt the same way. The group encourages, supports, accompanies, motivates, lifts you up, and drives you to achieve and challenge yourself to fulfil the goals”* G4-P3
	Psychological impact of the COVID-19 pandemic	*“This time we all had to exercise together made you forget about the COVID-19 situation, of not being able to see friends or family. You think about other things, like how strong you feel, or how you are attempting an exercise that you couldn’t do it before, and now you could lift my arm higher”* G1-P3
		*“I used to walk from the balcony to the dining room, then from the dining room to the bedroom... I was heartbroken by the fact that I couldn’t go out in public because of the pandemic. I can tell you that this group exercise time has been a gamechanger for me”* G2-P1

## Data Availability

The data that support the findings of this study are available on request from the corresponding author. The data are not publicly available due to privacy or ethical restrictions.

## Supplementary Materials

The following supporting information can be downloaded at: https://www.mdpi.com/article/10.3390/jpm12030356/s1, Table S1: Interview protocol for focus groups.
