# Supplementary material for: Breast Cancer Patients’ Experiences with Online Group-Based Physical Exercise in a COVID-19 Context: A Focus Group Study
Source: J Pers Med. 2022 Feb 26;12(3):356. doi: 10.3390/jpm12030356 (PMC8950528; doi:10.3390/jpm12030356)
Supplement: Supplementary file 1 [file jpm-12-00356-s001.zip › jpm-1583463-supplementary.pdf]

Table S1. Interview protocol for focus groups.

Stages for the Interview	Topics	Examples
Introductio n	Purpose of the study	My college and I are participating in a study to better understand breast cancer patients' experience towards physical exercise in a COVID-19 context. We believe that an online group-based exercise programme during the cancer treatment could be beneficial for cancer-related side effects and improve physical and psychological well-being, and your experiences may be useful in exploring the benefits and drawbacks of it.
	Objectives	Carry out and publish research based on the experience of women with breast cancer who did an online group-based exercise programme during their cancer treatment in a COVID-19 context.
	Ethical considerations	Our conversation will be recorded solely for research purposes in order to carry out our research. Just keep in mind that participation is entirely voluntary, and you can opt out at any time. Everything said during this focus group will be kept strictly confidential, anonymised, and available only to the research team.
	Verbal and formal consent	Granted if the participant agreed verbally and signed the formal consent.
Developm ent	Focus group grid	How would you describe your participation in the online group-based sport program during your treatment? What factors were the most important to you, and why?<br>How did it make you feel to follow the online exercise in streaming, and what benefits or drawbacks do you see from your perspective?<br>And, in terms of the side effects of your treatment, how do you think or feel that this program has helped you, and do you think it could have been harmful in the first place?<br>How has the COVID-19 situation affected your day-to-day life, both mentally and physically? Has this type of exercise made a difference in those situations, and if so, how?<br>Could you please tell us about your day-to-day life before you started the program? Do you believe your routine has changed as a result of your participation?<br>For those of you who did sports on your own before joining the program, what do you think the differences are? What do you think is best and why?
Closing	Final questions	Do you have anything else to add that might be relevant? Anything to clarify before we end?
	Acknowledge ments	Thank you for your time and interest. Certainly, your statements will be useful for the research.
	Consideration s	Please, let us know if you need anything else.<br>Once the study is finished, we will send you a copy.
Translation	Retro-translations	Interview statements will be translated by one bilingual researcher to English. Then, another bilingual research will back-translate them to Spanish and compared with the original transcripts to maintain their accuracy.
